# GateView: A Multi-Omics Platform for Gene Feature Analysis of Virus Receptors within Human Normal Tissues and Tumors

**DOI:** 10.3390/biom14050516

**Published:** 2024-04-25

**Authors:** Yang Sun, Zi-Liang Huang, Wen-Xin Chen, Yi-Feng Zhang, Hao-Tian Lei, Qiao-Juan Huang, Zhao-Rong Lun, Liang-Hu Qu, Ling-Ling Zheng

**Affiliations:** MOE Key Laboratory of Gene Function and Regulation, State Key Laboratory for Biocontrol, School of Life Sciences, Sun Yat-sen University, Guangzhou 510275, China; suny335@mail2.sysu.edu.cn (Y.S.); hziliang@mail2.sysu.edu.cn (Z.-L.H.); wc645@cornell.edu (W.-X.C.); zhangyf326@mail2.sysu.edu.cn (Y.-F.Z.); leiht@mail2.sysu.edu.cn (H.-T.L.); huangqj@mail.sysu.edu.cn (Q.-J.H.); lsslzr@mail.sysu.edu.cn (Z.-R.L.)

**Keywords:** virus, receptor, tissue, expression, function

## Abstract

Viruses are obligate intracellular parasites that rely on cell surface receptor molecules to complete the first step of invading host cells. The experimental method for virus receptor screening is time-consuming, and receptor molecules have been identified for less than half of known viruses. This study collected known human viruses and their receptor molecules. Through bioinformatics analysis, common characteristics of virus receptor molecules (including sequence, expression, mutation, etc.) were obtained to study why these membrane proteins are more likely to become virus receptors. An in-depth analysis of the cataloged virus receptors revealed several noteworthy findings. Compared to other membrane proteins, human virus receptors generally exhibited higher expression levels and lower sequence conservation. These receptors were found in multiple tissues, with certain tissues and cell types displaying significantly higher expression levels. While most receptor molecules showed noticeable age-related variations in expression across different tissues, only a limited number of them exhibited gender-related differences in specific tissues. Interestingly, in contrast to normal tissues, virus receptors showed significant dysregulation in various types of tumors, particularly those associated with dsRNA and retrovirus receptors. Finally, GateView, a multi-omics platform, was established to analyze the gene features of virus receptors in human normal tissues and tumors. Serving as a valuable resource, it enables the exploration of common patterns among virus receptors and the investigation of virus tropism across different tissues, population preferences, virus pathogenicity, and oncolytic virus mechanisms.

## 1. Introduction

In recent years, global pandemics caused by viruses have inflicted significant damage on human society, with the recent outbreak of COVID-19 being a stark example. The ability of viruses to initiate infection relies on specific receptor molecules located on the surface of target cells. However, variations in the sequences, expressions, and cellular distribution of these receptor molecules among individuals play a pivotal role in determining host susceptibility, viral tropism, and the severity of diseases [1]. For instance, a natural mutation in the *CCR5* gene hinders the entry of HIV strains into immune cells, thereby providing protection against infection for affected individuals [2].

The causative agent of COVID-19, SARS-CoV-2, utilizes ACE2 as its entry receptor, and *ACE2* is expressed in various tissues, including the lungs, intestines, kidneys, testes, and heart. As a result, many COVID-19 cases have presented symptoms such as pneumonia, diarrhea, vomiting, kidney failure, and cardiac injury [3,4]. Studies have confirmed a positive correlation between *ACE2* expression and in vitro infection with SARS-CoV [5]. Additionally, the genetic characteristics of *ACE2* are associated with varying susceptibility to COVID-19 among different populations [6,7,8]. In a separate analysis, Huang et al. investigated the expression profiles of the SARS-CoV-2 co-receptor NRP1 in cancer patients and identified a link between susceptibility to SARS-CoV-2 infection and the presence of tumors [9]. However, it remains unclear whether these findings are specific to the coronavirus receptor or represent broader patterns observed in virus receptors across humans.

Currently, more than 200 viruses capable of infecting humans have been identified, yet less than half of them have known receptor molecules. Comprehensive research on the overall patterns of these virus receptor molecules is still lacking. In recent years, a range of multi-omics sequencing technologies have emerged, encompassing genomics, transcriptomics, proteomics, and epitranscriptome. Additionally, single-cell transcriptomics and spatial transcriptomics technologies have been developed, offering higher resolution in depicting gene expression levels. Some studies have employed single-cell sequencing to analyze the distribution characteristics of SARS-CoV-2 receptor genes [10]. However, there is currently no multi-omics data resource available for over a hundred human virus receptors, posing challenges for conducting joint analyses and discovering their shared characteristics.

**Figure 1:**
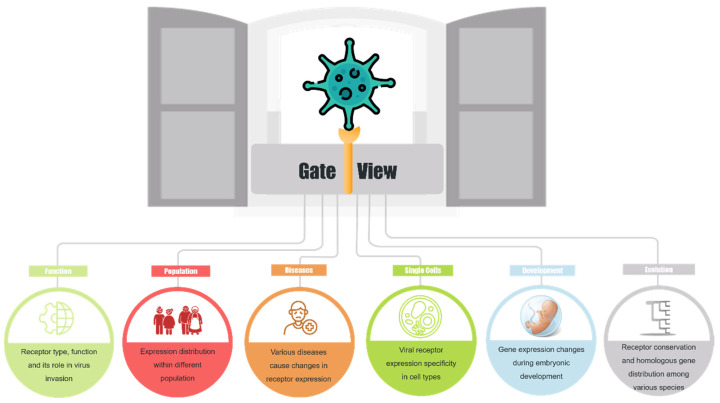
The overview of GateView.

To address this gap, GateView (https://rna.sysu.edu.cn/gateview/index.php; 1 July 2021) was established as a comprehensive platform for analyzing the features of virus receptors within human tissues and single cells (Figure 1). Building upon this foundation, the multi-omics characteristics of various virus receptors under different populations and conditions, including gender, age, tissue, organs, and physiological or pathological states, have been explored (Table S1). In summary, GateView provides valuable data resources for studying virus–host interactions and elucidates the overarching attributes of receptor molecules. This knowledge enhances the understanding of virus invasion mechanisms and their potential applications in disease treatment.

## 2. Materials and Methods

### 2.1. Data Collection

The interaction between viruses and receptors, as well as the corresponding annotation, primarily relies on the integration of data from various sources (Figure S1). The main database used for this purpose was ViralZone (https://viralzone.expasy.org/; accessed on 12 April 2020) [11]. Additionally, the viralReceptor database (http://www.computationalbiology.cn:5000/viralReceptor; accessed on 15 April 2020) was also utilized [12]. In order to verify and supplement the data, references from NCBI Taxonomy (https://www.ncbi.nlm.nih.gov/Taxonomy/Browser/wwwtax.cgi; accessed on 11 April 2020), ViralHostDB (https://www.genome.jp/virushostdb/; accessed on 25 April 2020) [13], and PubMed (https://www.ncbi.nlm.nih.gov/pubmed/; accessed on 10 April 2020) were consulted. The homologous genes of each receptor gene in 21 representative species were initially downloaded from NCBI’s HomoloGenes (Build 68) (https://ftp.ncbi.nih.gov/pub/HomoloGene/last-archive/; accessed on 15 May 2020). The homologous genes of the human ACE2 gene were derived from 11 species (Table S2).

The transcriptomes of normal tissue were collected using Bulk-seq GTEx Release V8 (https://gtexportal.org/home/downloads/; accessed on 8 May 2020) [14]. The transcriptome data for cancer tissue were obtained from TCGA (https://www.cancer.gov/tcga; accessed on 18 June 2020), while the transcriptome data for smoker lung tissue were sourced from GEO (https://www.ncbi.nlm.nih.gov/geo/; accessed on 4 July 2020) dataset GSE1643 [15]; Th cell line expression data were obtained from the Human Protein Atlas (https://www.proteinatlas.org/; accessed on 18 August 2020) [16]; The transcriptome data for fetal development were retrieved from BioProject (https://www.ncbi.nlm.nih.gov/bioproject/; accessed on 20 July 2020) PRJNA270632 [17], and the transcriptome data for placental development were obtained from GEO datasets GSE9984 [18], GSE37901 [19] and GSE22490 [20].

The single-cell transcriptome data were obtained from various sources, including the COVID-19 Cell Atlas (CCA, https://www.COVID19cellatlas.org/; accessed on 1 July 2020) [21], Human Cell Atlas (HCA, https://www.humancellatlas.org/; accessed on 13 July 2020) [22], the 10X Genomic website (https://www.10xgenomics.com/cn/datasets; accessed on 3 August 2020), and GEO. RNA modification sites were collected from RMBase (https://rna.sysu.edu.cn/rmbase/index.php; accessed on 12 August 2020) [23], and DNA variation data from dbSNP (https://www.ncbi.nlm.nih.gov/snp/; accessed on 15 August 2020) [24].

After integrating data from multiple sources and assigning unified identifiers, non-receptor genes were excluded, and further analysis of receptor gene multi-omics data were stored in the GateView database (Table S3).

### 2.2. Data Processing, Statistics and Web Implementation

#### 2.2.1. Analysis of Receptor Expression Levels

Initially, sequencing samples from 30 tissues were obtained from GTEx data for the bulk-seq transcriptome analysis. The mean expression of viral receptor genes in each tissue was then calculated, followed by logarithmic transformation of the mean values. Differential gene expression analysis was performed using limma (v.3.46.0) (https://bioconductor.org/packages/limma/, 17 April 2024) [25] and edgeR (v.3.31.1) (https://bioconductor.org/packages/edgeR/, 17 April 2024) [26]. The Benjamini-Hochberg adjusted *p*-values were reported. For co-expression analysis, Pearson correlation coefficients and significance levels for each gene pair were computed across all sequencing samples within each tissue.

Based on the annotation files provided by GTEx, the samples were categorized into two groups based on gender (male and female) and two age groups (older and younger, with a cutoff age of 60 years). The differential gene expression analysis was conducted using limma, and heatmaps were generated using the Heatmap function from ComplexHeatmap (v.2.6.2) (https://bioconductor.org/packages/ComplexHeatmap/, 17 April 2024) [27].

#### 2.2.2. Single-Cell Sequencing Data Analysis

For the analysis of single-cell transcriptome data, two software packages were employed: Scanpy (v1.6.0) (https://github.com/theislab/Scanpy, 17 April 2024) [28] in Python and Seurat (v3.2.0) (http://www.r-project.org/, 17 April 2024) [29] in R. These packages were used for quality control, preprocessing, cell type annotation, and visualization. In cases where the dataset was obtained from COVID-19 Cell Atlas (CCA), this study utilized the original subtype annotations provided by the authors [30]. For other datasets, cell subtype annotations were performed using the SingleR package (v1.3.6) (https://bioconductor.org/packages/SingleR/, 17 April 2024) [31], with the Human Primary Cell Atlas dataset serving as a reference. Manual corrections were made as necessary.

In the co-expression analysis, the expression matrix for each cell type was extracted initially, and genes with expression levels >0 were retained. The Spearman correlation coefficient was then calculated between receptor genes and all human genes in the matrix. P-values were adjusted to control the false discovery rate (FDR) using the Benjamini-Hochberg method. Gene pairs with an FDR < 0.05 were retained. Additionally, a gene set enrichment analysis, with 15 gene sets for annotation, was conducted for each cell type across all tissues.

#### 2.2.3. Evolutionary Conservation Analysis and Phylogenetic Analyses

PhastCons scores, ranging from 0 to 1, indicate the probability of conservation for a single nucleotide across multiple species [31]. To assess the conservation of a transcript, the phastCons scores for all exons of that transcript were averaged across 100 vertebrate species. Human data were obtained from UCSC (hg38.phastCons100way.bw). Human gene transcript information from GENCODE version 39 was utilized, with only “basic” annotated transcripts retained. The Linux software bigWigAverageOverBed (v2) (https://hgdownload.soe.ucsc.edu/, 17 April 2024) was employed to calculate the average phastCons scores across all exons of these retained transcripts, excluding any empty values.

The sequences were pairwise aligned using the Needleman-Wunsch algorithm on the EBI web server to obtain similarity and identity, with parameters set as -datafile EBLOSUM62 -gapopen 10.0 -gapextend 0.5 -endweight -endopen 10.0 -endextend 0.5. [32]. Multiple sequence alignment for key regions was performed using the ClustalW algorithm in MEGA11 [33]. The phylogenetic tree was inferred using the Maximum Likelihood method and JTT matrix-based model [34]. The tree with the highest log likelihood (−10,750.05) was presented. Initial trees for the heuristic search were automatically obtained by applying Neighbor-Join and BioNJ algorithms to a matrix of pairwise distances estimated using the JTT model, and then selecting the topology with the superior log likelihood value. This analysis involved 11 amino acid sequences, with a total of 1039 positions in the final dataset. Evolutionary analyses were conducted using MEGA11 [33].

#### 2.2.4. Cancer Data Analysis

In the analysis of cancer data, the values of transcript fragments per kilobase of exon per million mapped reads (FPKM) were compared between tumor tissues and their corresponding normal tissues. This differential gene expression analysis was conducted using the limma package. For gene set enrichment analysis, the GSEA function from clusterProfiler (v.3.18.1) (https://bioconductor.org/packages/clusterProfiler/, 17 April 2024) was employed [35]. Initially, gene–cancer pairs were sorted based on the log2-transformed differential fold change of each gene across cancer types. A list of virus types corresponding to each receptor gene, referred to as TERM2GENE, was curated. Lastly, the gseaplot2 function from enrichplot was used to generate enrichment distribution plots [35].

#### 2.2.5. Database Architecture

The web interface was developed using a suite of frontend libraries. Bootstrap controlled the layout and styling of the web pages, while jQuery enables dynamic interactions on the web pages. The dataTable library presented the analysis results in the form of data frame. Additionally, Highchart was employed to visualize the analysis results through divers formats. We guarantee that GateView will be maintained for a minimum of 5 years.

## 3. Results

### 3.1. The Main Features of GateView: Using ACE2 as an Example

#### 3.1.1. GateView Demostrates the Correlation between Viruses and Receptor Molecules

**Figure 2:**
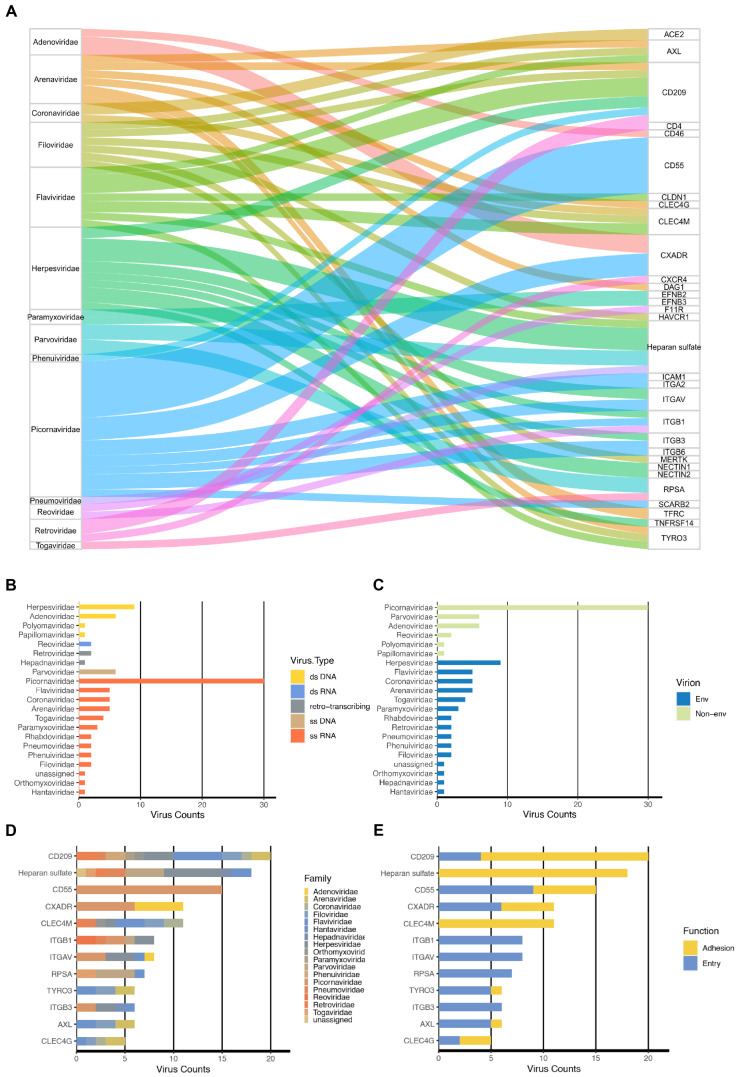
Viruses and receptors distribution. (**A**) The correspondence between major virus families and their receptor molecules. Each square on the left side displays the name of a virus family collected in GateView, with the size of the square representing the number of viruses it contains. Each square on the right side shows the protein names of the receptor molecules bound by these viruses, with the size of the square indicating how many viruses can bind to them. The lines in the middle indicate the binding relationships between viruses and receptor molecules. The figure only displays receptor molecules that bind to at least two viruses, removing those that serve as receptors for only one virus. (**B**) Number and types of viruses across various virus families. (**C**) Number of viruses within different virus families, with color-coded indication of envelope presence. (**D**) The number of corresponding viruses in different receptors, showing only those receptors interact with at least five types of viruses, with the color indicating the family to which the corresponding virus belongs. (**E**) The number of corresponding viruses in different receptors, and the color indicates the role of that receptor in different viruses.

**Figure 3:**
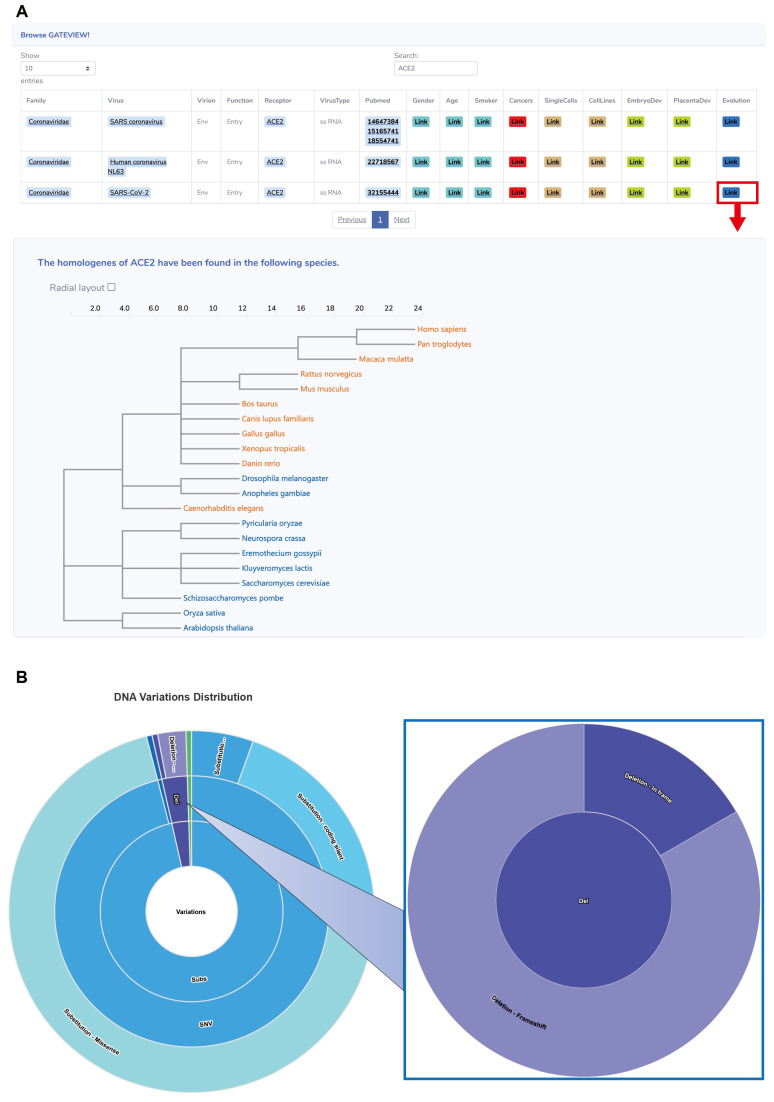
Search results for the gene *ACE2* on GateView. (**A**) GateView Browse page search results for the ACE2 receptor, highlighting the distribution of its homologous genes. (**B**) Search results on the GateView DNA variations page for DNA variation type distribution in the receptor gene *ACE2*.

GateView catalogues 220 pairs of interactions between human viruses and cellular receptors, involving a total of 91 human viruses, encompassing 21 virus families and 74 cellular receptors (Figure 2A). To categorize these viruses, the Baltimore classification system was applied, segregating them into five distinctive groups: double-stranded DNA viruses (dsDNA), single-stranded DNA viruses (ssDNA), double-stranded RNA viruses (dsRNA), single-stranded RNA viruses (ssRNA), and retroviruses (Figure 2B,C). Further analysis revealed numerous many-to-one or one-to-many relationships between virus families and receptors. For instance, CD209 serves as the cell receptor for ten virus families. Most of these families employ it as an adhesion receptor, whereas the remainder utilize it as an entry receptor. GateView’s Browse page enables users to explore the 220 pairs of relationships between viruses and receptors. For example, a search for ACE2 illustrates its primary role as the entry receptor for viruses within the Coronaviridae family. To access specific result pages, users can click on the corresponding link button in the search results. In our investigation of the distribution of homologous genes of virus receptors among 21 model organisms, selecting the link page in the Evolution section revealed that *ACE2* homologous genes are primarily found in animals, including *Homo sapiens*, *Pan troglodytes*, *Macaca mulatta*, *Rattus norvegicus*, *Mus musculus*, and others. However, these homologous genes are absent in representative plant and bacterial species (Figure 3A). The evolutionary tree constructed based on these homologous gene sequences effectively reflects the evolutionary relationships among species (Figure S2). To delve deeper into the sequence variations within homologous genes, pairwise sequence alignment was conducted, focusing on the binding region with the S protein of SARS-CoV-2. It is known that three specific regions on the amino acid sequence of human ACE2 form the binding site of the SARS-CoV-2 S protein, specifically R1: 30–41 (12 AAs), R2: 81–84 (4 AAs), and R3: 353–357 (5 AAs) [36]. Notably, these segments exhibit complete identity across the three representative primate species (Figure S2). Conversely, in the other two representative mammalian species (*Canis lupus familiaris* and *Bos taurus*), sequence variations are observed in the R1 and R2 regions, while R3 remains identical to that of primates. In the rodent representative species (*Rattus norvegicus* and *Mus musculus*), variations are present in the R1-R3 regions, with a single AA mutation at position 353 from K to H in the R3 region. These sequence variances highlight the highly conserved nature of the key binding domain of ACE2 with the SARS-CoV-2 S protein in primates, suggesting that SARS-CoV-2 may require specific prerequisites for spread in other primate species. The impact of variant sites in other animals on SARS-CoV-2 invasion warrants further investigation through experimental and structural biology studies.

#### 3.1.2. Multiple Types of DNA Variations in *ACE2* Gene across Different Cancer Types

Through the integration of cancer genomic mutation data, DNA variation events within virus receptor genes were extensively examined across different cancer types. These identified DNA variation types, including substitutions, deletions, and duplications, were further classified into subcategories. For instance, the substitution category was further stratified into SNV (Single Nucleotide Variant) and DNV (Dinucleotide Variants), each further stratified into more precise classifications. These hierarchical relationships were visually depicted using an interactive donut chart. Specifically, the analysis revealed a total of 201 DNA variations within the *ACE2* gene across 33 distinct cancer types. Among these variations, the majority (96.5%) were substitutions, 3% were deletions, and a sole event represented a duplication. By clicking on the Del part, users can explore the distribution of deletion subtypes, where 83.3% correspond to frameshift deletions and 16.7% to in-frame deletions (Figure 3B). Furthermore, a detailed table presents users with comprehensive information on mutation types, mutation sites, nucleotide changes, COSMIC ID, and associated references within a receptor gene, providing a comprehensive understanding of potential mutations.

#### 3.1.3. *ACE2* Expression Exhibits Gender or Age Differences in Specific Tissues

This study delineated the relationship between receptor expression levels in tissues and viruses’ tissue tropism. Moreover, it revealed that variations in receptor expression among different genders and age groups are correlated with the susceptibility of populations. The TissueMap tool was developed to visually present these expression variances. It allows users to select a particular virus receptor gene and examine its distribution across tissues and subpopulations.

**Figure 4:**
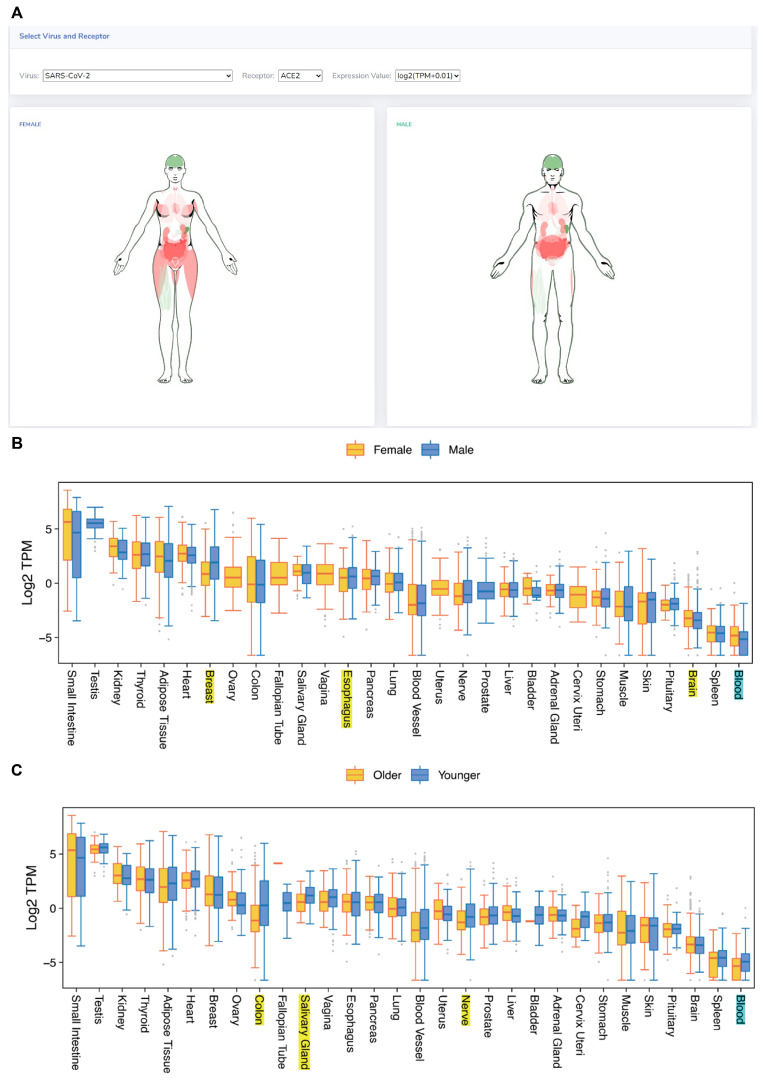
Distribution of receptors in various tissues and populations. (**A**) Tissue map page in GateView. (**B**) Expression levels of *ACE2* in different tissues across genders. (**C**) Expression levels of *ACE2* in different tissues across age groups. Noted annotations represent significant differences (*p*-value < 0.05) in tissue samples, with blue highlighting the presence of both gender and age disparities in blood samples.

Using GateView’s TissueMap page, the expression level of *ACE2* across diverse tissues was specifically investigated (Figure 4A). The small intestine exhibited the highest *ACE2* expression, consistently observed across genders and age groups (Figure 4B,C). While *ACE2* expression did not manifest significant gender disparities across most tissues, considerable variations were evident in female blood and brain tissues, where ACE2 expression markedly surpassed that of males. Conversely, *ACE2* expression was significantly lower in the esophagus and breast among females. Regarding age disparities, *ACE2* displayed substantial variations in 13% of the analyzed tissues. Particularly, the expression of *ACE2* in the blood, colon, nerve, and salivary glands of elderly individuals notably decreased compared to younger individuals (Figure 4B). *ACE2* expression in the blood exhibited disparities both in gender and age.

#### 3.1.4. *ACE2* Displays Distinct Co-Expression Patterns in Diverse Cell Types

**Figure 5:**
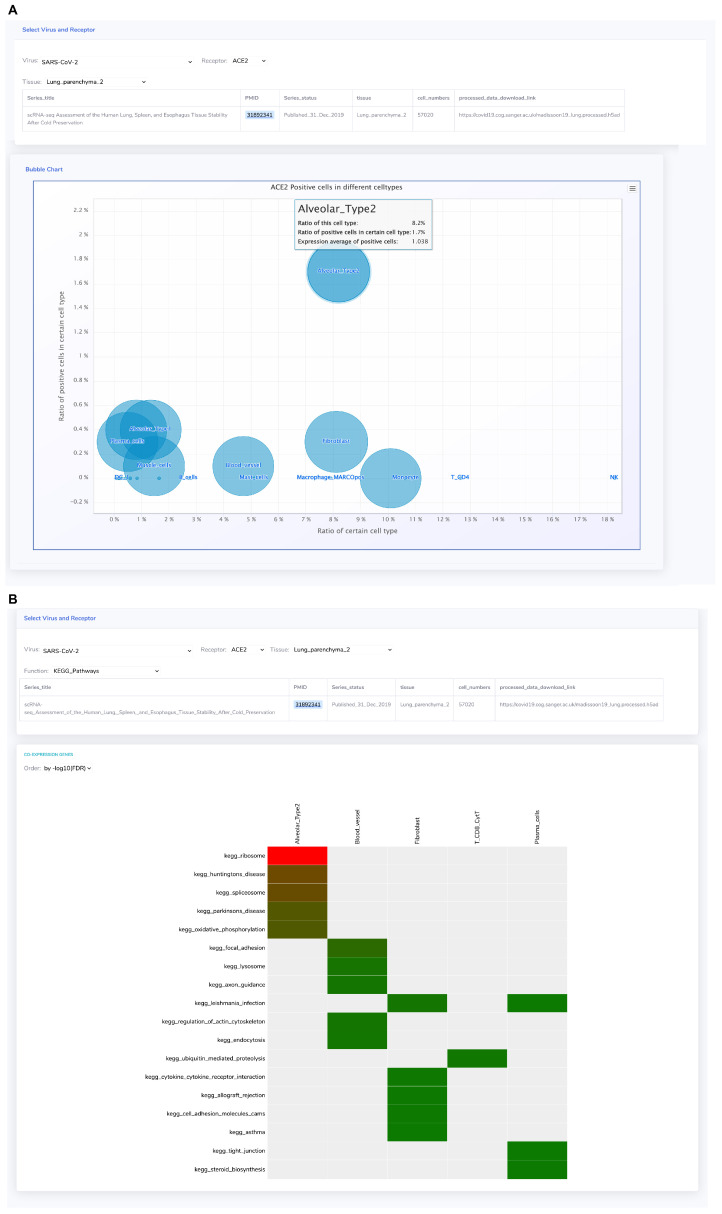
Expression patterns of receptor genes in different cell types. (**A**) Bubble plot depicting the distribution of *ACE2* in cell types within lung tissue. (**B**) Heatmap of KEGG pathways enriched with genes significantly co-expressed with *ACE2* in lung cells.

Tissue samples comprise various cell types, each expressing receptors in a cell type-specific manner. Particularly, multiple studies have consistently highlighted the specific expression of *ACE2* in Alveolar Type 2 (AT2) cells within the lung, making these cells the primary targets for SARS-CoV-2 infection [37,38]. In this investigation, GateView utilized 39 single-cell sequencing datasets from 24 tissues to construct a receptor distribution bubble chart, demonstrating the distribution of receptor genes across distinct cell types within a two-dimensional space (Figure 5A). This chart offers insights into the distribution of *ACE2* across 25 cell types identified in lung tissue. The chart’s horizontal axis signifies the proportion of each cell type within the tissue, while the y-axis represents the proportion of ACE2-positive (ACE2+) cells among each cell type. The dot size on the chart corresponds to the mean expression level of *ACE2* in ACE2+ cells. The visualization highlights that while AT2 cells constitute a relatively minor proportion (8.2%) of the total cell types present in lung tissue, they exhibit the highest proportion of ACE2+ cells (1.7%), distinguishing them from other cell types that exhibit proportions less than 0.5%. Thus, the unique positioning of AT2 cells in the upper right corner of the receptor distribution bubble chart distinctly identifies them among other cell types.

An analysis of genes exhibiting significant co-expression with the receptor gene in each cell type was conducted to explore their enriched functions and pathways. To assess their functional associations, enrichment analysis was performed across 15 functional sets, resulting in the development of the “Single-cell Co-expression Gene Function Map” tool (Figure 5B). The analysis revealed that in AT2 cells, genes co-expressed with *ACE2* were significantly enriched in the ribosome pathway (*p*-value = 10^−63.5^, FDR = 10^−61.3^). However, in the blood vessel cells, genes significantly co-expressed with *ACE2* were enriched in the focal adhesion pathway (*p*-value = 10^−10.9^, FDR = 10^−8.66^). These findings highlight distinct biological functions associated with receptor genes across different cell types (Figure 5A).

### 3.2. Comprehensive Analysis of Diverse Human Virus Receptor Molecules

#### 3.2.1. Comparative Analysis of Virus Receptors and Other Membrane Proteins

More than 19,847 distinct proteins are situated on the surface of human cells. However, viruses specifically target a limited subset of these molecules, employing them as receptors. To discern the distinctive attributes of these receptor molecules, an analysis was performed by contrasting their sequence and expression levels with those of other membrane proteins. For this purpose, an exhaustive list of membrane proteins was assembled from the Human Protein Atlas (HPA) database and categorized into two groups: human virus receptors and non-receptor membrane proteins.

**Figure 6:**
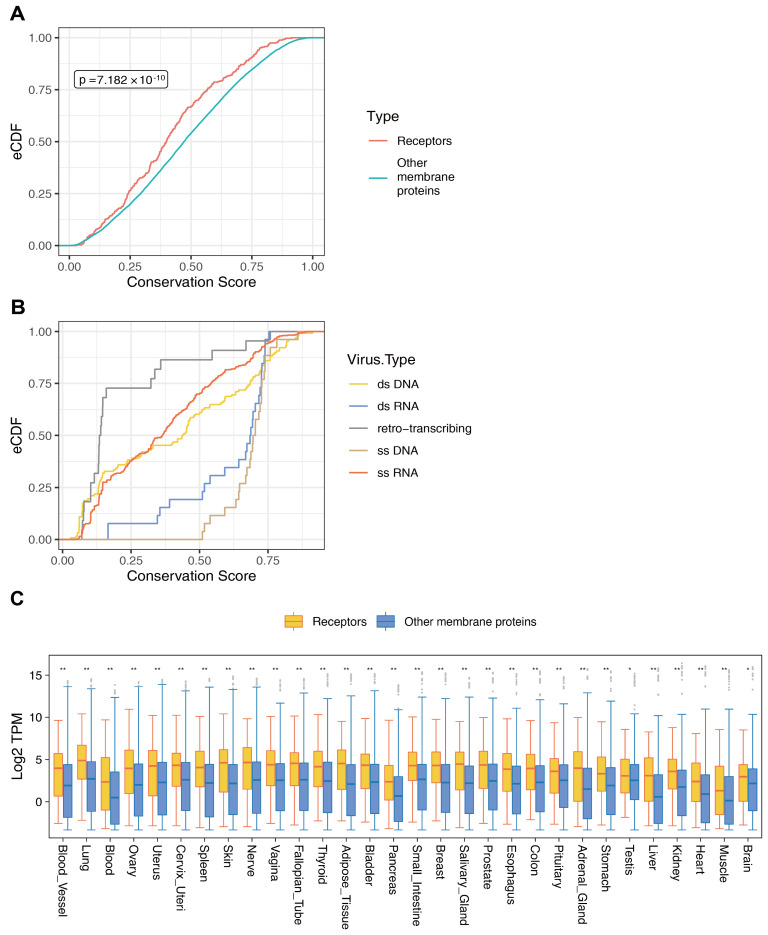
Comparison of features between virus receptor molecules and other membrane proteins. (**A**) Comparative analysis of evolutionary conservation between viral receptors and other membrane proteins across 100 vertebrate species. (**B**) Comparative analysis of evolutionary conservation of viral receptors for different types of viruses across 100 vertebrate species. (**C**) Comparative analysis of the expression patterns of viral receptors and other membrane proteins in various tissues or organs. * indicates a *p* value less than 0.05, ** indicates a *p* value less than 0.01, and blank indicates insignificance.

To investigate the evolutionary conservation of receptor genes, the sequence conservation of membrane proteins across humans and 99 other vertebrate species was examined. Employing an empirical cumulative distribution function plot, the mean conservation scores of receptor molecules were compared with those of other membrane proteins. The findings demonstrated that virus receptors generally exhibited lower conservation scores compared to other membrane proteins (Figure 6A). This observation implies that virus receptors exhibit a reduced level of evolutionary conservatism when compared to other membrane proteins, with noticeable discrepancies observed among distinct species.

Additionally, the correlation between virus types and the conservation of their corresponding receptor molecules was analyzed. The outcomes revealed notable variations in the conservation of receptor molecules among different virus types. Specifically, receptor molecules linked to single-stranded DNA (ssDNA) viruses and double-stranded RNA (dsRNA) viruses generally demonstrated elevated conservation scores compared to receptor molecules associated with other virus categories. Conversely, receptor molecules of retroviruses exhibited the lowest overall conservation (Figure 6B).

Subsequently, the expression levels of viral receptors and other membrane proteins were compared across 30 distinct human tissues. The findings revealed that viral receptors generally exhibited higher average expression levels compared to other membrane proteins across tissues (Figure 6C). Specifically, in some tissues or organs, the average expression level of viral receptors exceeded those of other membrane proteins by more than 1.5 times. These outcomes imply a tendency for viruses to preferentially target molecules exhibiting higher expression levels as their receptors.

#### 3.2.2. Receptor Molecules Demonstrate Pronounced Tissue Specificity

**Figure 7:**
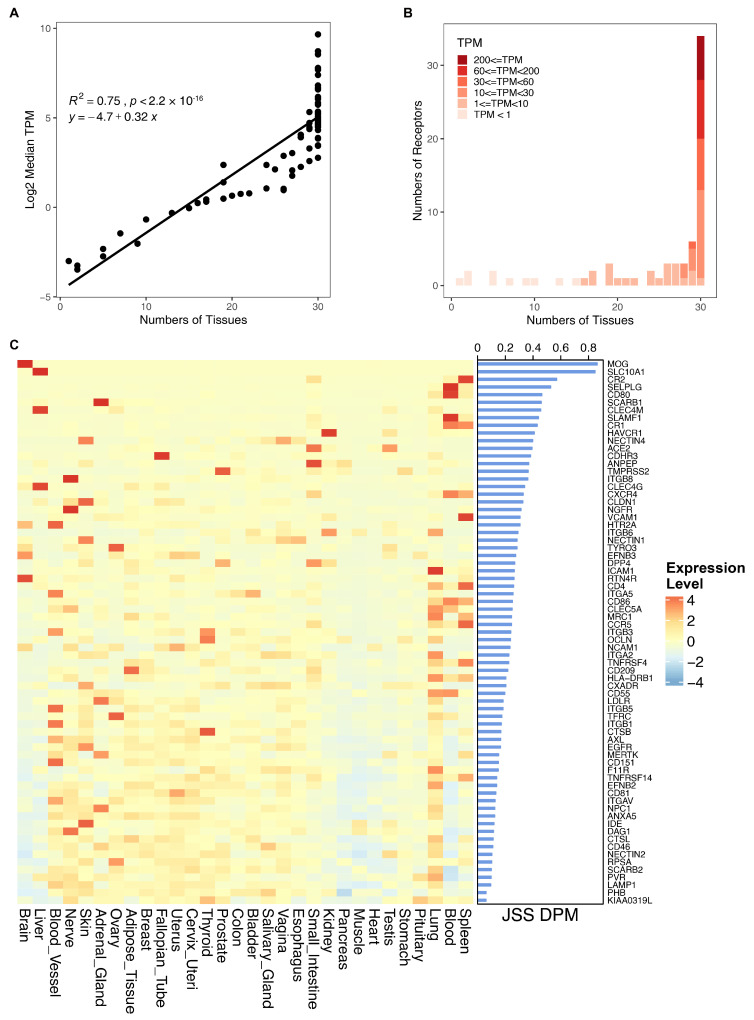
Distribution of viral receptor expression. (**A**) Correlation between viral receptor expression and the number of tissues expressed. (**B**) Number of viral receptors and the tissues in which they are expressed. (**C**) Left panel: expression levels of viral receptors in each tissue. Right panel: distribution of Jensen–Shannon specificity dispersion scatter.

Considering the well-established tissue tropism exhibited by viruses, it becomes crucial to ascertain whether their receptors exhibit attributes indicative of tissue-specific expression. To explore this, an analysis of virus receptors across different tissues was initially conducted. The median expression levels of receptor genes across 30 human normal tissues were compared with the number of tissues in which they exhibited expression (greater than or equal to 1 Transcript Per Million). The results revealed a significant correlation between the median expression level of receptors and the number of tissues where they were expressed, signifying those receptors with higher median expression tend to manifest across multiple tissues, while those with lower median expression are only expressed in specific tissues (Figure 7A). Moreover, the investigation unveiled that over half of the receptor molecules manifested expression in all 30 tissues (Figure 7B), suggesting their pivotal involvement in fundamental cellular functions and the necessity for their expression across diverse tissues.

Given the widespread expression of receptor molecules across multiple tissues, it becomes pivotal to explore whether their expression distribution remains uniform throughout all tissues or if it tends to be notably elevated in specific ones. Figure 7C presents the expression levels and tissue-specificity scores of receptors across 30 tissues. The results show that 31% of receptors exhibit marked tissue-specific expression characteristics (JSS DPM greater than 0.3). Notably, the receptor MOG for the rubella virus manifests considerably higher expression levels in the brain compared to other tissues. Similarly, the receptors SLC10A1 for hepatitis B and hepatitis D viruses showcase notably heightened expression exclusively within the liver tissue. Comparative analysis of different tissues reveals that 21% of virus receptors tend to be highly expressed in lung tissue (Z-score greater than 2), followed by spleen (15%) and blood vessels (13%). These findings indicate that while virus receptors are generally expressed in various tissues, their expression levels in specific tissues are significantly higher than in others, demonstrating clear tissue-specific expression characteristics.

#### 3.2.3. Receptor Molecule Expression Levels Differ among Populations

**Figure 8:**
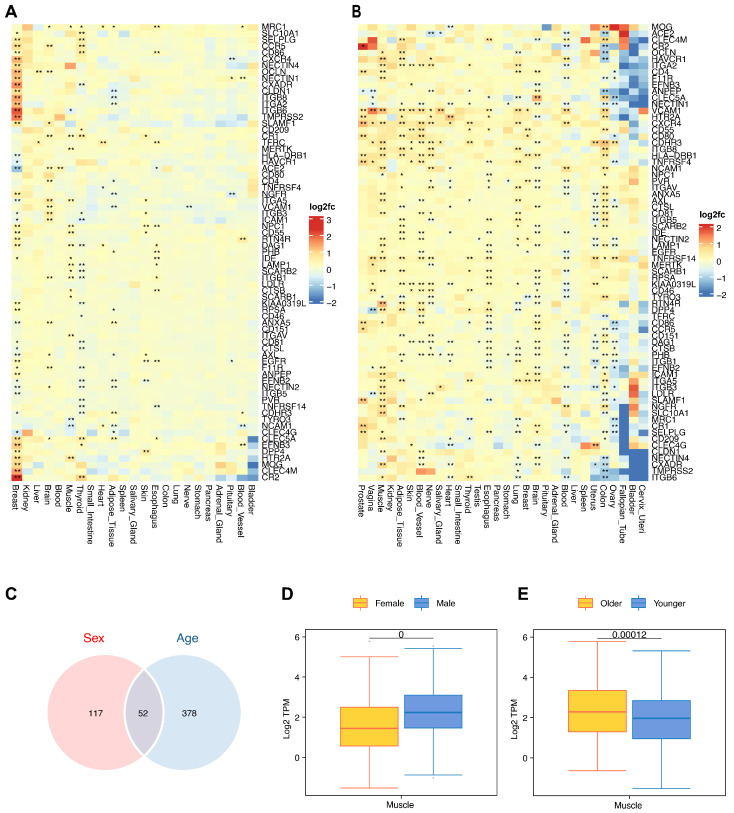
Expression distribution of viral receptors. (**A**) Differential results for viral receptors in each tissue between females and males. (**B**) Differential results for viral receptors in each tissue between individuals over 60 years of age and those under 60 years of age. Color indicates the fold difference. * indicates a *p* value less than 0.05, ** indicates a *p* value less than 0.01, and blank indicates insignificance. (**C**) Receptor–tissue pairs displaying significant differences in both sex and age within the same tissue. (**D**) Sex differences of NCAM1 in muscle. (**E**) Age differences of NCAM1 in muscle.

For the majority of tissues or organs, virus receptors do not exhibit substantial gender-related differences in expression. However, approximately 15% of virus receptors manifest notably elevated expression levels in the female thymus. Remarkably, receptors such as CR2 for human gamma-herpesvirus 4 and ITGB6 for human alpha-herpesvirus 1 exhibit significantly higher expression in the female thymus compared to male thymus tissue, with expression levels approximately five times and four times higher, respectively (Figure 8A). In most tissues, it was revealed that receptor expression levels differed significantly between the under-60 age group and those over 60. Additionally, a distinct pattern emerges, suggesting elevated expression of virus receptors in the tissues and organs of older individuals (Figure 8B).

A total of 49% of the receptor molecules exhibited significant disparities in both age and gender within the same tissue (Figure 8C). Notably, the receptor molecule for the rabies virus, NCAM1, demonstrated the most substantial fold change. Its expression level in male muscle tissue was 1.51 times higher than in female muscle tissue (Figure 8D), whereas in the muscle tissue of older individuals, it was 1.46 times higher compared to younger individuals’ muscle tissue (Figure 8E).

These findings show that while the expression levels of most virus receptors are consistent across genders, notable difference emerge across different age populations.

#### 3.2.4. Receptor Molecule Expression Levels Are Generally Dysregulated in Cancer

**Figure 9:**
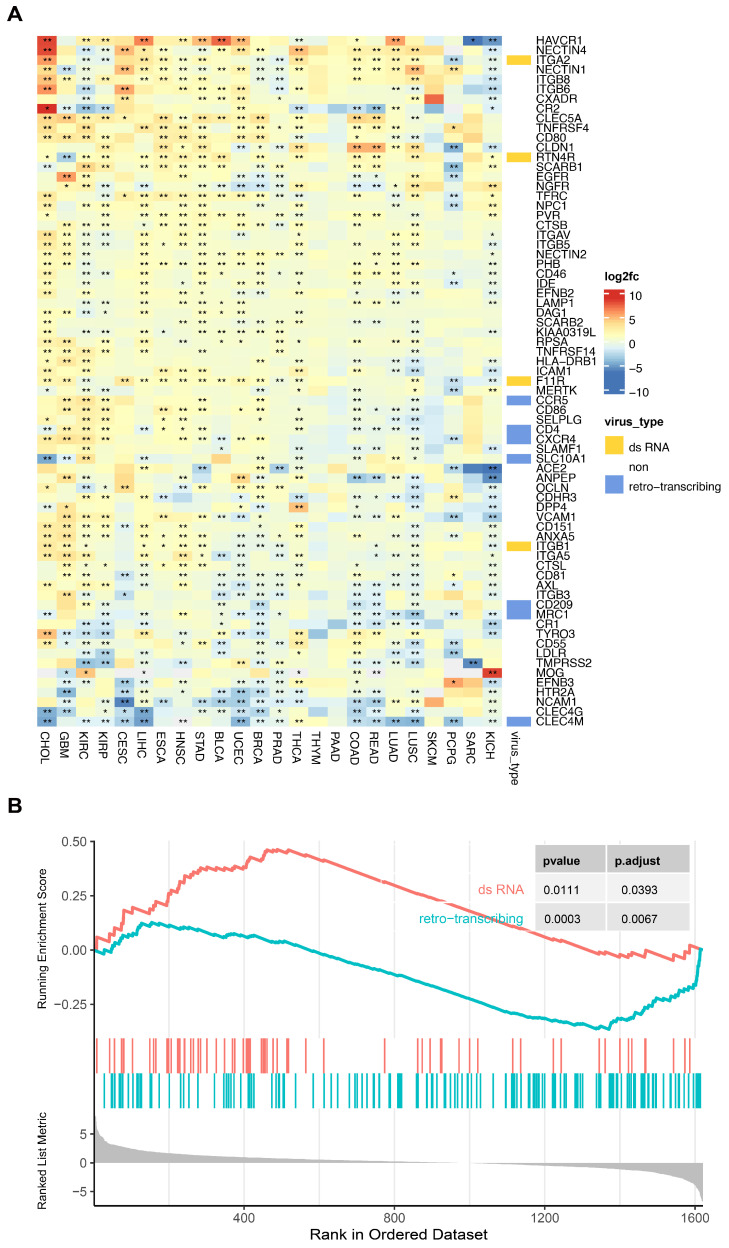
Differential expression of viral receptors in cancers. (**A**) Comparative analysis of viral receptors in tumor tissue as opposed to normal histology for each specific cancer type. * indicates a *p* value less than 0.05, ** indicates a *p* value less than 0.01, and blank indicates insignificance. (**B**) Enrichment analysis demonstrating the association between virus types and their corresponding viral receptors.

Research has highlighted a clear association between specific cancers and viral infections. For instance, long-term carriage of Hepatitis B Virus (HBV) can increase the risk of developing liver cancer, and Human Papillomavirus (HPV) is considered one of the primary causative factors of cervical cancer. In this study, an analysis of the expression differences of virus receptors in tumor compared to normal tissues was conducted. The results revealed substantial dysregulation in the expression of most virus receptors in cancerous tissues (Figure 9A). Notably, certain virus receptors exhibited remarkably high-fold differences. For instance, the adhesive receptor CR2 for Herpesviridae displayed an upregulation of over 400 times in cholangiocarcinoma (CHOL), while the receptor MOG for the Rubella virus showed an upregulation of over 300 times in kidney chromophobe (KICH) cancer.

To further examine potential patterns in receptor molecule variations in cancer, the correlation between virus types and alterations in their respective receptor molecules within the context of cancer was analyzed. The results demonstrate that virus receptors for dsRNA viruses displayed a significant upregulation across various cancers (normalized enrichment score, NES, of 1.58), whereas virus receptors for retroviruses were predominantly downregulated (NES of −1.58) (Figure 9B).

## 4. Discussion

It is widely recognized that the tissue tropism of viruses is intricately linked to the distribution patterns of their receptor molecules. In the present investigation, a captivating observation was made that the expression levels of human virus receptor molecules exhibit considerable variation among different tissues, despite their general expression throughout various tissues. This divergence in expression levels may account for the virus’s tropism to infect specific tissues or organs. The findings suggest that a significant proportion of virus receptors exhibit a notable degree of tissue specificity. This outcome can be interpreted in two ways. Firstly, high receptor expression alone is not sufficient for virus invasion; it also depends on the specific cell type and its microenvironment. Secondly, in tissues with heightened receptor expression, the virus may enter a latent or slow replication state, resulting in minimal tissue damage and clinical symptoms. Notably, studies have documented that the SARS-CoV-2 virus can persist in feces longer than in throat swabs [39], suggesting the possibility of viral latency in the intestines. Moreover, there is compelling evidence pointing towards the invasion of testicular tissue by COVID-19, as certain patients have reported testicular pain and potential impairment in male fertility [40]. These significant observations highlight the critical need for diligent surveillance of tissues exhibiting distinct expression patterns of receptor molecules.

Canas reported notable disparities in COVID-19 symptomatology based on gender and age [41]. Following SARS-CoV-2 infection, male patients commonly exhibit symptoms such as shortness of breath, fatigue, chills, and fever, whereas females tend to experience symptoms like loss of smell, chest pain, and cough. Moreover, among the elderly population, there is a heightened prevalence of symptoms including diarrhea. While the mechanisms elucidating these disparities in symptom presentation are intricately complex, the study identified significant gender and age discrepancies in the primary receptor for SARS-CoV-2, ACE2. These findings suggest that variations in virus receptors may potentially underlie the heterogeneity of post-infection symptoms observed across different demographic groups.

Our study revealed a significant decrease in the expression level of ACE2 in various tissues among older individuals, including blood, colon, bile, and salivary glands. This finding is consistent with previous reports that have established a correlation between aging and reduced *ACE2* expression [42,43]. In theory, reduced expression of receptor molecules could potentially result in a lower infection rate and less severe disease. However, contrary to this expectation, current research indicates that individuals aged 60 and above display a higher susceptibility to infection compared to their younger counterparts [44]. This incongruity suggests the involvement of other factors influencing susceptibility to viral infections. Consequently, further comprehensive investigations are warranted to shed light on the underlying population differences in viral infections.

In addition to comparing expression levels, the study also assessed the evolutionary conservation of receptor molecules compared to non-receptor membrane proteins. The findings reveal that receptor molecules exhibit lower conservation when contrasted with non-receptor membrane proteins. The evolution of virus receptors is influenced by two distinct opposing evolutionary pressures. Firstly, negative selection operates to preserve the cellular functions of these molecules. Concurrently, positive selection is fueled by the ongoing host–virus arms race [31,45,46]. Wang et al. demonstrated that virus receptors undergo stronger positive selection compared to non-virus receptor genes with similar functions, a finding that aligns with the results [47]. Our analysis of various virus families indicates that retrovirus receptor molecules exhibit the lowest conservation, significantly lower than receptor molecules for other virus types. As this observation has not been previously reported, the underlying mechanism remains unclear.

Although the development and progression of cancer are influenced by various internal and external factors, infection with oncogenic viruses is one significant factor, with approximately 13% of diagnosed cancers in 2018 being associated with infections from human papillomavirus (HPV), hepatitis B virus (HBV), hepatitis C virus (HCV), and Epstein–Barr virus (EBV) [48,49,50]. In this study, by analyzing the differential expression of various human virus receptors in different types of cancer, dysregulation of multiple virus receptors in cancer was observed. The upregulation of these virus receptor expressions in cancer cells suggested that viral infections may partially inhibit the survival of cancer cells. Some studies have reported that individuals with cancer may be more susceptible to SARS-CoV-2 [51] infection or more likely to develop severe COVID-19. In summary, additional research is required to comprehensively comprehend the correlation between tumors and infectious diseases. The GateView database serves as a valuable resource for data and analytical tools, facilitating investigations into the intricate association between viral receptors and the onset and progression of cancer, and supporting research into therapeutic strategies.

The blood–brain barrier (BBB) is a critical structure that tightly regulates the movement of molecules, ions, and cells between the blood and the brain [52,53]. In the context of viral infections, BBB dysfunction has been observed in conditions such as viral encephalitis, where the virus can directly infect and damage the endothelial cells. Additionally, in systemic viral infections, inflammatory mediators can indirectly disrupt the BBB. SARS-CoV-2 can interact with the BBB through various mechanisms, including direct interactions with brain endothelial cells and indirect effects mediated by inflammation, clotting, and hypoxia [53]. Using single-cell sequencing technology to analyze the BBB after viral infection can detect the effects of viral infection on cell composition, receptor molecules, and validation factors, thus studying the relationship between BBB disruption and viral infection. The method proposed in GateView is helpful for studying the distribution of virus receptors in different cell types. In the future, we will collect scRNA-Seq data of BBB after viral infection to further expand our database.

## 5. Conclusions and Outlook

Virus receptors play a pivotal role in facilitating the initial recognition and binding of viruses to infected cells, thereby influencing susceptibility to virus infection. In this study, the GateView platform was introduced as a comprehensive collection of diverse human virus receptors. Leveraging multi-omics data, an extensive analysis was conducted to elucidate the distinctive characteristics of these virus receptors.

The investigation encompassed several common characteristics of virus receptor molecules, including: lower sequence conservation compared to other cell membrane surface protein genes and higher expression levels compared to other cell membrane surface protein genes. Although virus receptors are expressed in most tissues, they tend to be more highly expressed in specific tissues (not necessarily the target tissues of the virus). Most virus receptors show differences in expression levels among populations of different ages, but there are very few gender differences. Significant dysregulation of virus receptor expression in cancer tissues, especially for dsRNA and retrovirus receptors.

We developed the GateView database to store and display the data on these virus receptor features (Table S1). The data resources and analytical tools provided by this study offer invaluable resources for advancing our understanding of human viruses and enhancing preventive strategies against infectious disease pandemics.

In recent years, several specialized virus receptor databases, such as VThunter, viralReceptor, and EVIHVR, have been established [12,54,55], which have compiled a wealth of information on receptor-related characteristics. These databases provide important data resources for researchers from different perspectives, facilitating systematic analysis of the characteristics and functions of virus receptors. In the future, further expanding the database content and improving data coverage and accessibility could help present a comprehensive landscape of virus receptors.

Delving into the structural characteristics of virus receptors and their interaction mechanisms with viral particles could help elucidate the molecular basis of virus infection. Additionally, understanding how different types of viruses utilize host receptors for infection and transmission could provide new insights for developing targeted antiviral strategies.

In summary, virus receptor research is an important and promising field. Leveraging the rich database resources, future breakthroughs in virology, immunology, and drug development can be anticipated.

## Data Availability

All data supporting the findings of this study can be found within the paper. Additional data supporting the findings of this study are available from the corresponding author upon request.

## Supplementary Materials

The following supporting information can be downloaded at: https://www.mdpi.com/article/10.3390/biom14050516/s1. Table S1. Comparative Analysis of Features between GateView and Other Databases. Table S2. The results of the comparison between human ACE2 protein and its homologs. Table S3. The list of source databases for the original data integrated in GateView. Figure S1. The workflow of Gateview. Figure S2. The comparison between the SARS-CoV-2 virus S protein binding regions of the human ACE2 protein and the proteins encoded by homologous genes of the human ACE2 gene in different species. Left: The Maximum Likelihood tree. Right: Multiple sequence alignment of the key regions. Different residues are highlighted in red.
