# Supplementary material for: GateView: A Multi-Omics Platform for Gene Feature Analysis of Virus Receptors within Human Normal Tissues and Tumors
Source: Biomolecules. 2024 Apr 25;14(5):516. doi: 10.3390/biom14050516 (PMC11118183; doi:10.3390/biom14050516)
Supplement: Supplementary file 1 [file biomolecules-14-00516-s001.zip › biomolecules-2937730-supplementary.pdf]

biomolecules
MDPI

Supplementary Materials

GateView: A Multi-Omics Platform for Gene Feature Analysis of Virus Receptors within Human Normal Tissues and Tumors

Yang Sun †, Zi-Liang Huang †, Wen-Xin Chen †, Yi-Feng Zhang, Hao-Tian Lei, Qiao-Juan Huang, Zhao-Rong Lun, Liang-Hu Qu * and Ling-Ling Zheng *

MOE Key Laboratory of Gene Function and Regulation, State Key Laboratory for Biocontrol, School of Life Sciences, Sun Yat-sen University, Guangzhou 510275, China; suny335@mail2.sysu.edu.cn (Y.S.); hziliang@mail2.sysu.edu.cn (Z.-L.H.); wc645@cornell.edu (W.-X.C.); zhangyf326@mail2.sysu.edu.cn (Y.-F.Z.); leiht@mail2.sysu.edu.cn (H.-T.L.); huangqj@mail.sysu.edu.cn (Q.-J.H.); lsslzr@mail.sysu.edu.cn (Z.-R.L.)

* Correspondence: lssqlh@mail.sysu.edu.cn (L.-H.Q.); zhengll33@mail.sysu.edu.cn (L.-L.Z.); Tel.: +86-20-84112517 (L.-L.Z.)

† These authors share equal senior authorship.

Table S1. Comparative Analysis of Features between GateView and Other Databases.

Data_items	VirusReceptor	Viralzone	GateView
Family	✔	✔	✔
Taxonomy	✔	✔	✔
Virion	✔	✔	✔
Receptor	✔	✔	✔
Function		✔	✔
Viral protein		✔	✔
Type	✔	✔	✔
Literature	✔		✔
Receptor detailed information	✔		✔
**Improved features**			
Population (Sex, Age)			17382 samples / 30 tissue
Smoking history			33 sample / Lung
Cancer			10327 samples / 33 type
Single Cells			28 samples / 24 tissue / 1061868 cells
Cell Lines			64 strains
Basic disease			13 samples / 8 type
Embryo Development			35 samples / 6 tissues
Placenta Development			3 samples
Evolution			74 homology groups / 21 species

Table S2. The results of the comparison between human ACE2 protein and its homologs.

Accession ID	Taxonomy	Identity	Similarity
NP_068576.1	*Homo sapiens*	100.00%	100.00%
XP_016798468.1	*Pan troglodytes*	99.01%	99.38%
NP_001129168.1	*Macaca mulatta*	94.91%	97.52%
NP_001158732.1	*Canis lupus familiaris*	83.48%	91.80%
NP_001019673.2	*Bos taurus*	80.99%	90.56%

NP_001012006.1	*Rattus norvegicus*	82.48%	90.06%
NP_001123985.1	*Mus musculus*	82.11%	89.57%
XP_416822.3	*Gallus gallus*	65.44%	79.34%
XP_002938293.2	*Xenopus tropicalis*	55.67%	69.68%
NP_001007298.1	*Danio rerio*	55.73%	69.76%
NP_001024453.1	*Caenorhabditis elegans*	20.32%	35.01%

Table S3. The list of source databases for the original data integrated in GateView.

Database	Website	Download time
ViralZone	https://viralzone.expasy.org/	2020.04.12
viralReceptor	http://www.computationalbiology.cn:5000/viralReceptor	2020.4.15
ViralHostDB	https://www.genome.jp/virushostdb/	2020.04.25
HomoloGenes	https://ftp.ncbi.nih.gov/pub/HomoloGene/last-archive/	2020.05.15
GTEx	https://gtexportal.org/home/downloads/	2020.05.08
TCGA	https://portal.gdc.cancer.gov/	2020.06.18
CCA	https://www.covid19cellatlas.org/	2020.07.01
HCA	https://www.humancellatlas.org/	2020.07.13
10X Genomic	https://www.10xgenomics.com/cn/datasets	2020.08.03
RMBase	https://rna.sysu.edu.cn/rmbase/	2020.08.12
dbSNP	https://www.ncbi.nlm.nih.gov/snp/	2020.08.15
Human Protein Atlas	https://www.proteinatlas.org/	2020.08.18

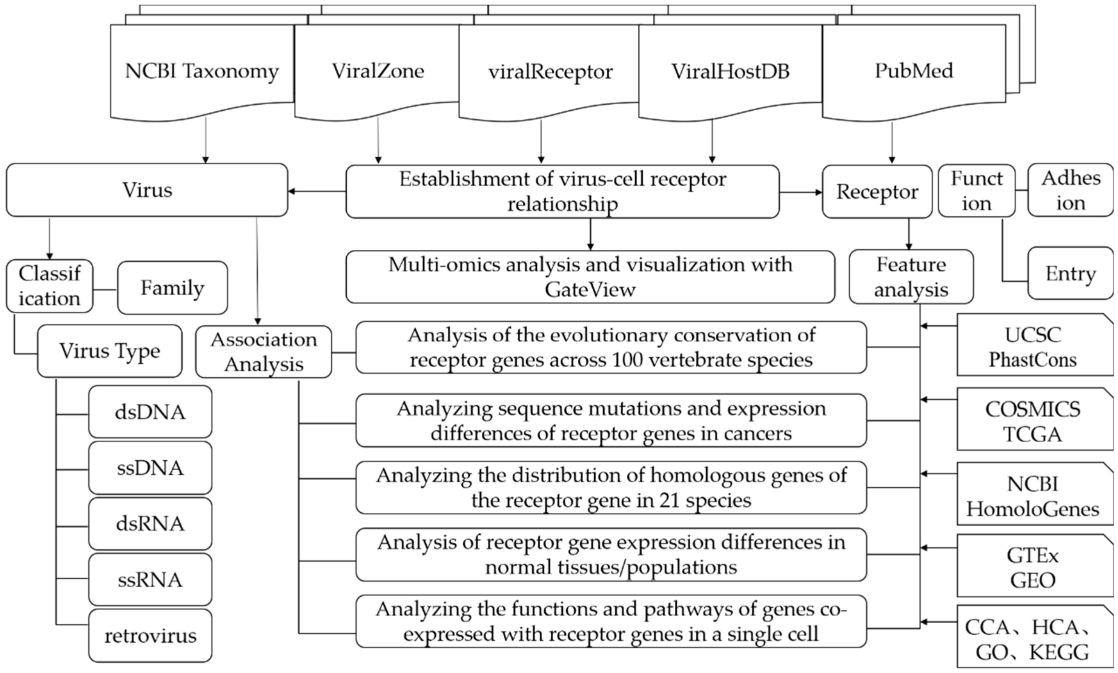

Figure S1. The workflow of Gateview.

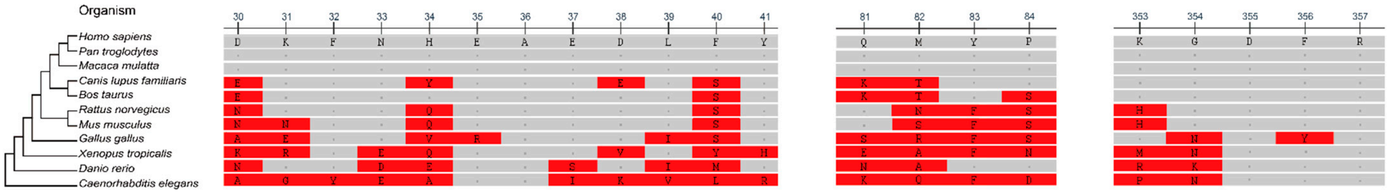

Figure S2. The comparison between the SARS-CoV-2 virus S protein binding regions of the human ACE2 protein and the proteins encoded by homologous genes of the human ACE2 gene in different species. Left: The Maximum Likelihood tree. Right: Multiple sequence alignment of the key regions. Different residues are highlighted in red.
